# Binding-Site Match Maker (BSMM): A Computational Method for the Design of Multi-Target Ligands

**DOI:** 10.3390/molecules25081821

**Published:** 2020-04-16

**Authors:** Jinming Zhou, Jian Hui Wu

**Affiliations:** 1 Key Laboratory of the Ministry of Education for Advanced Catalysis Materials, Department of Chemistry, Zhejiang Normal University, 688 Yingbin Road, Jinhua 321004, China; 2 Drug Discovery and Innovation Center, College of Chemistry and Life Sciences, Zhejiang Normal University, 688 Yingbin Road, Jinhua 321004, China; 3 Segal Cancer Center, Montreal, QC H3T 1E2, Canada; 4 Lady Davis Institute for Medical Research, Sir Mortimer B. Davis-Jewish General Hospital, McGill University, 3755 Cote-Ste-Catherine, Rd., Montreal, QC H3T 1E2, Canada; 5 Department of Oncology, McGill University, 3755 Cote-Ste-Catherine, Rd., Montreal, QC H3T 1E2, Canada

**Keywords:** multi-target ligand, drug design, geometric hashing, similar binding site

## Abstract

Multi-target ligand strategies provide a valuable method of drug design. However, to develop a multi-target drug with the desired profile remains a challenge. Herein, we developed a computational method binding-site match maker (BSMM) for the design of multi-target ligands based on binding site matching. BSMM was built based on geometric hashing algorithms and the representation of a binding-site with physicochemical (PC) points. The BSMM software was used to detect proteins with similar binding sites or subsites. In particular, BSMM is independent of protein global folds and sequences and is therefore applicable to the matching of any binding sites. The similar sites between protein pairs with low homology and/or different folds are generally not obvious to the visual inspection. The detection of such similar binding sites by BSMM could be of great value for the design of multi-target ligands.

## 1. Introduction

Many signaling networks in mammalian cells are likely to be wired with redundant pathways, such that optimal therapeutic interventions can be achieved through perturbing multiple nodes of the networks [1]. Most modern searches for new drugs take place within the terrain of the “One-drug one-target” paradigm. Such a reductional approach is fruitful but it does not exploit the network complexity and pathway redundancy. It is now generally accepted that activity at a single receptor is insufficient for a complex disease involving multiple factors such as diabetes, neurodegenerative syndromes, cardiovascular diseases, or cancer. Studies that seek to achieve the synergistic effects by one agent against multiple targets (referred to as multi-target ligands) or a combination of multiple individual agents have been emerging [2,3,4]. For example, tyrosine kinase inhibitor imatinib inhibits BCR-ABL, PDGF receptor and c-kit simultaneously [5]. Although many currently marketed drugs act via multiple targets, the discovery of their multi-targeting properties is usually serendipitous [4]. For example, in addition to cyclooxygenase-2, aspirin is found to interact with phospholipase A2, phospholipase C, and IKK kinase [6,7,8]. Celecoxib, an anti-inflammatory drug that was designed to inhibit COX-2, has unexpected nanomolar inhibition potency against carbonic anhydrase (CA) [9]. Compounds that demonstrate significant activity against targets irrelevant to the disease might lead to toxic side effects; therefore, multi-target ligands must be selective. A structure-based approach for the rational design of multi-target ligand is highly desirable. 

The current predominant technique for the generation of multi-target ligands is based on the selective ligand through a combination of pharmacophores, which could be classified into the following cases: (i) The pharmacophores are joined together by a cleavable or non-cleavable linker. (ii) The pharmacophores are partially overlapped by taking advantage of structural commonalities. (iii) The pharmacophores are highly overlapped and are incorporated into the same chemical scaffold. The molecular modeling has been employed to design a multi-target ligand by performing pharmacophore analysis [10,11]. Detecting targets with similar binding sites might provide another approach for the design of the multi-target ligands. To date, a series of methods/algorithms for evaluating binding-site similarity have been reported. For the methods implemented in pvSOAR [12], CavBase [13] and SiteEngine [14], the binding-site was represented by specific features, such as the physiochemical pseudocentres, triangle mesh surface representations of protein functional sites on Connolly surface, as well as the sequence fragment. For SiteBase [15,16], the binding-site was represented by atoms, and the comparison of the binding-site was done using all-against-all. Recently, several studies combining the atom type and the physiochemical pseudocentres were reported [17,18,19]. The geometric hashing algorithms were widely applied in the process of the binding-site matching [14,15,16,20,21]. Some other algorithms, such as the graphic theory [22] and knowledge-based potentials [23] were also utilized for the binding-site matching. Most of these binding-site similarity studies are intended to computationally predict protein function by the binding-site similarity to known proteins [14,15,16,20,21], or to predict new protein targets for a drug [17]. In the present work, based on geometric hashing algorithms, we have developed a modeling program to automatically identify protein pairs that share similar binding-sites, independent of protein global fold and sequence. The software is referred to as the binding-site match maker (BSMM) and it is applicable to the rational design of multi-target ligands. 

## 2. Materials and Methods

### 2.1. Data Set

The following two sets of protein-ligand complexes were collected from the protein data bank (PDB, www.pdb.org) [24]: (i) set A has 15 ligands, consisting of 33 protein pairs that are individually with the sequence identity from 19% to 35%, and the full list of protein-ligand complexes for set A was shown as Table S1 (Supplemental Materials); (ii) set B has 52 ligands, consisting of 77 protein pairs that are individually with the sequence identity < 14%, and the full list of protein-ligand complexes for set B was shown as Table S2 (Supplemental Materials).

### 2.2. Representation of the Binding-Site 

**Figure 1:**
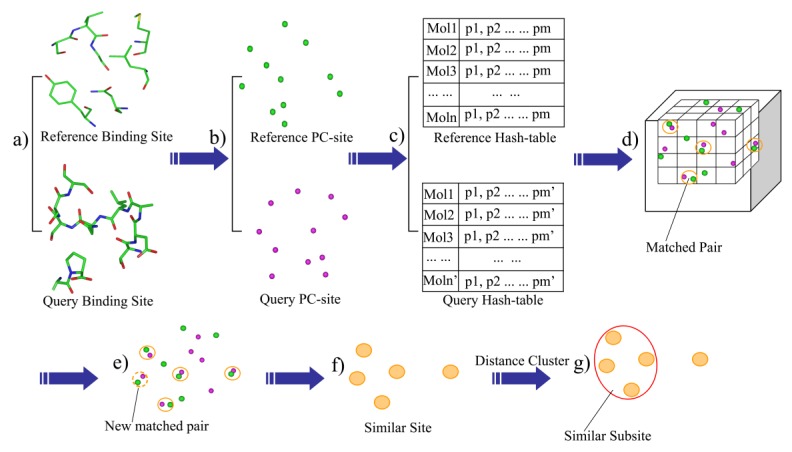
The flowchart of BSMM.

Given a protein-ligand complex, residues within 6.0 Å of the bound ligand were extracted and defined as the binding-site (Figure 1a). The outputted site within 6 angstroms of the ligand could provide the whole binding site and avoid the redundant residues which would add the computational consumption (7 angstroms). The heavy-atom model was used in the distance calculations. The binding-site was then described as a set of the physicochemical type points, such as the hydrogen acceptor (HA), hydrogen donor (HD), mixed hydrogen acceptor and donor (HAD), aliphatic hydrophobic (ALI) and aromatic properties (ARO). Next, the physicochemical sites (PC-site) were generated by extracting the physicochemical type points within a defined distance (6 Å) of the bound ligand (Figure 1b).

### 2.3. Generation of the Binding-Site Hashing File

BSMM was developed based on geometric hashing algorithms, which have been adopted in the protein matching [14,16,20,21]. First, a series of hashing files were generated according to the points set in the PC-site: given n description points of a binding-site, randomly choose the three non-collinear points among them to form a triangle, order the three sides of the triangle by length, make the point face the shortest side as P1, mark the point face the longest side as P3, and the remaining side as P2. A coordinate system is then defined based on this reference triangle (P1-P2-P3). A transformed matrix is derived based on the new defined coordinate system, which can transform the coordinates of the description points of the binding-site into the new coordinate system (see the Supplementary Materials for details). To generate the hash files of a binding-site, all possible triangles were checked and the point–point distance cutoffs were employed to define the upper and lower boundaries of the triangle side so that the number and the size of reference triangles can be controlled. For each reference triangle: (1) calculate the new coordinate transformed matrix according to the triangle; (2) transform the coordinates of the points in the binding-site into the new coordinate system, which are collectively defined as a model, and save it to the hash file; (3) index each model by the point properties of the three points and the lengths of the three sides in the reference triangle. Thus, for each binding-site, the hash file can have many models, and each of these models represents the same binding-site in a unique coordinate system (Figure 1c).

### 2.4. The Process of Binding-Site Matching 

**Figure 2:**
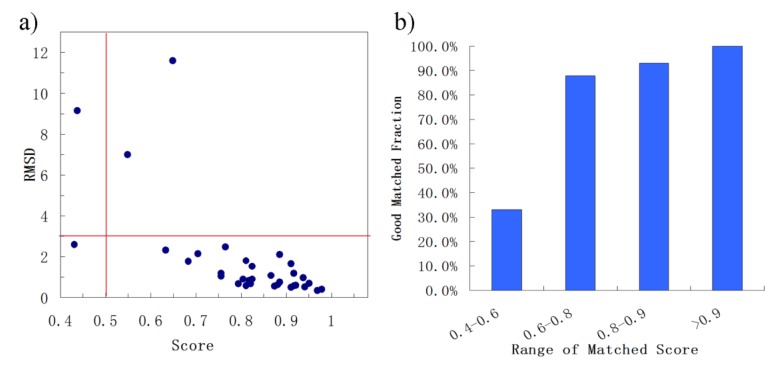
The summary of the matched results of BSMM for data set A: given the good matching should be RMSD < 3.0 Å, the rate of good matching for data set A is 91% (30/33); carefully checking the pairs which the matched RMSD is > 3.0 Å found the binding sites of the pairs matched well, and the high RMSD due to the different binding mode of the ligands. (**a**) the correlation between the ligand RMSD and the the matched score; (**b**) the good matching rate in different range of matched score.

**Figure 3:**
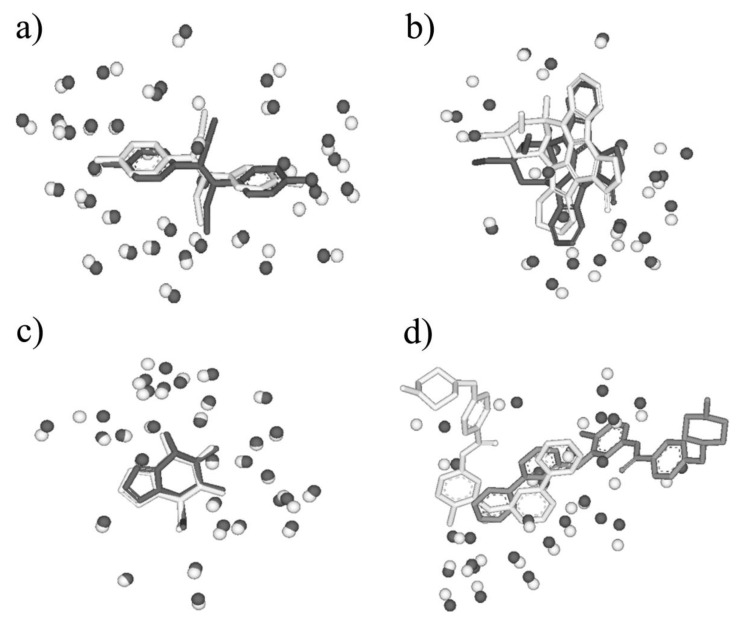
(**a**) The matched binding sites for Diethylstilbestrol between ERRγ (in white, PDB entry: 1S9P) and ERα (in black, PDB entry: 3ERD) by BSMM (Sequence Identity: 32%, Mscore: 0.875); (**b**) the matched binding sites for Staurosporine between CDK2 (in white, PDB entry: 1AQ1) and EGFR (in black, PDB entry: 2ITQ) by BSMM (Sequence Identity: 19%, Mscore: 0.811); (**c**) the matched binding sites for Glucoimidazole between Beta-galactosidase (in white, PDB entry: 2CEQ) and Beta-glucosidase (in black, PDB entry: 2CES) by BSMM (Sequence Identity: 28%, Mscore: 0.969). (**d**) the matched binding sites for imatinib between c-Kit (in white, PDB entry: 1T46) and Syk (in black, PDB entry: 2XBB) by BSMM (Sequence Identity: 32%, Mscore: 0.649).

The rules for maching PC points are shown in Table 1. The binding-site matching process is described as follows: Given the hash files of two binding-sites, one is defined as the reference and the other one is defined as the query. (1) One model of the reference hash table is then mapped into the matching box which is a cubic box with grids, and each point of the model is mapped into a grid according to its coordinates (Figure 1d); (2) Map one model of the query hash table into the matching box. If one point from the query hash table matches one point of the reference model in the same grid (the default grid bin width is 1.0 Å) and the PC properties of the two points are also matched, add a vote to the match vote (Figure 1d); (3) If the match vote is lower than the preset threshold (Set A: vote > 6, Set B: vote > 8), go back to step 2. Otherwise, perform the best least-square fit for all of the matched points of the two models. Next, all of the matched pairs that are within the preset distance cutoff (D_cutoff < 3.0 Å) are identified (Figure 1e), thus, the matched pairs are extended. (4) the matched score (M_score) is computed, and all of the matched pairs, which is defined as the similar site, is outputted (Figure 1f); (5) the cluster analysis of the matched points is performed, while the local matched score (L_score) is calculated. The largest family from the cluster analysis is defined as the similar subsite (Figure 1g); and (6) go back to step 2 and perform the next round of matching. 

### 2.5. Evaluation of Binding-Site Similarity

The Tanimoto score and Simpson score were utilized to evaluate the similarity of the two binding-sites as follows:

Mscore = Match_num/min(R_p_num, Q_p_num)

Lscore = L_Match_num/(LR_p_num + LQ_p_num – L_ Match_num)

Where, M_Score is the total Simpson score. Match_num is the total number of the matched points for the models of the two binding-sites, and R_p_num is the total number of the points from the reference binding-site (Figure 1b), whereas Q_p_num is the total number of the points from the query binding-site (Figure 1b). The L_Score is the local tanimoto Score. The L_Match_num is the number of the largest cluster. LR_p_num is the number of the points in the space that the largest family of the reference binding-site encloses. LQ_p_num is the number of the points in the space that the largest family of the query binding-site encloses. Generally, M_Score describes the similarity of the two binding sites, while L_Score evaluates the similarity of the sub sites.

## 3. Results and Discussions

### 3.1. The Evaluation of BSMM

The BSMM, written in perl and c++ programming language, is designed to match binding sites in three-dimensional space independent of protein folds and sequences. We have evaluated BSMM for its capability: (i) to match binding sites from the proteins with similar global folds; (ii) to match binding sites from proteins with different folds. We have utilized the bound ligand to evaluate the matched results. Specifically, given the crystal structures of two proteins (A and B) complexed with the same ligand (L), A/L and B/L, the binding sites of protein A and B are matched by BSMM and the matched points for both A and B are generated. To evaluate how well the match has been made by BSMM, the A/L crystal structure is aligned with the matched points of protein A, whereas the B/L crystal structure is aligned with the matched points of protein B. Next, RMSD between the L from the aligned A/L and the L from the aligned B/L is evaluated. If the binding modes of A/L and B/L are similar (If the binding modes are different, the RMSD value can not be used to evaluate the matching), a good matching of the two binding sites (means the binding site pair is similar) should produce small RMSD for ligand L. The sequence identity of the compared protein pair was also calculated through Clustalw2 web server (www.ebi.ac.uk/Tools/clustalw2/index.html) to describe the similarity of the global folds of them. In some case, even the total identified sequences will adopt the totally different folding structures. Q-score is a good measure of structural similarity of multiple protein in three dimensions [25]. Therefore, to check if the compared pairs adopt the similar global folds, the Q-Score for each protein pairs were also calculated and shown in Tables S1 and S2.

(1) Matching of the binding sites from proteins with the sequence identity > 19%

First, BSMM was employed to match binding sites of proteins with the sequence identity (19%–35%) in data set A. The results were reported in Table S1 (Supplementary Materials). In set A, there are 33 complexes pairs, consisting of 15 ligands. The complexes of each pair were bound by the same ligand, and the involved proteins were with the sequence identity (calculated by Clustalw2) of no less than 19%. As summarized in Figure 2, most of Mscores are higher than 0.7, with RMSDs of no more than 3.0 Å. Given that good matching should be RMSD < 3.0 Å, the rate of good matching for data set A is 91% (30/33). For the 30 pairs of which the RMSD values are no more than 3.0, both the ligand and binding site match well. Three examples were shown in Figure 3, which are the matched binding sites for Diethylstilbestrol between ERRγ and Erα (Figure 3a), the matched binding sites for Staurosporine between CDK2 and EGFR (Figure 3b), and the matched binding sites for Glucoimidazole between Beta-galactosidase and Beta-glucosidase (Figure 3c). In detail, of the two exceptions with the highest RMSD values, one is the pair of c-Kit and SYK (Figure 3d), of which the binding ligand is imatinib. The ligand RMSD for c-Kit vs. SYK is 11.59 Å as a result of BSMM, suggesting that the binding modes of imatinib in c-Kit and Syk are significant different. This is consistent with the experimental finding that imatinib binds to Syk in a cis-conformation that differs dramatically from the binding mode observed with c-Kit. Another two pairs of which the RMSD values were higher than 3.0 also indicated the difference of the binding mode of the ligand with the good match of the binding site. Therefore, it is important to verify that the matched RMSD value of the ligand could not evaluate the matching result of the binding site when the ligand bound two binding sites in a different mode, and the different binding mode of the same ligand is not occasional even for a high similar binding site as a result of data set A (about 13%). In general, when the proteins of the pair are in similar global folds, the matching results obtained by BSMM are highly credible.

(2) Matching of the Binding Sites from Proteins the Sequence Identity < 14%

**Figure 4:**
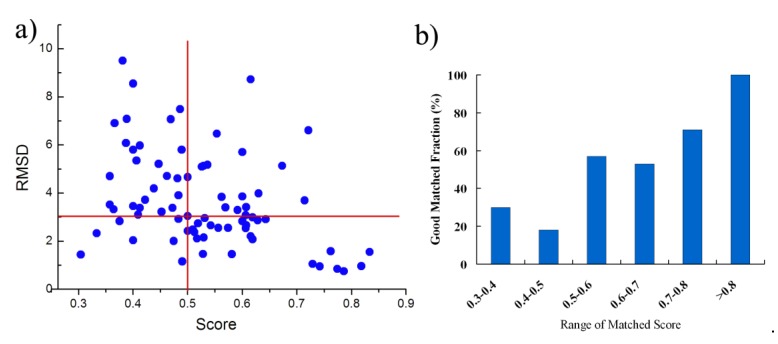
The summary of the matched results of BSMM for data set B: given the good matching should be RMSD < 3 **Å**, the rate of good matching for data set B is 44%; for Mscore > 0.5, the rate of good matching for data set B is 60%; the rate of good matching does not correlate to the sequence identity when the sequence identity < 12%. (**a**) the correlation between the ligand RMSD and the the matched score; (**b**) the good matching rate in different range of matched score.

As similarity between the binding sites from proteins with the sequence identity > 19% is generally high, it is easier to match the binding sites and BSMM performance very well in this case. Next, we have tested BSMM with binding sites of protein pairs in Set B, which include 129 complexes pairs, consisting of 77 ligands that are individually complex with proteins in a sequence identity < 14%. Furthermore, the Q-score was calculated to evaluate the structural similarity. The results are listed in Table S2 (Supplementary Materials) and are summarized in Figure 4. Given that good matching should be RMSD < 3.0 Å, the over-all rate of good matching for data set B is 44% (34/77); for Mscore > 0.5, the rate of good matching for data set B is 60% (27/45), and when the Mscore is higher, the rate of good matching is correspondingly higher (Figure 4b). Thus, when BSMM was applied to the pairs with different global folds, the good matching rate was dropped compared to that with similar folds. This was partially caused by that inaccuracy might be induced for the lower fold similarity of the binding site. However, when the Mscore is more than 0.5, the results indicate that BSMM is capable of matching binding sites from proteins with the sequence identity < 14%.

**Figure 5:**
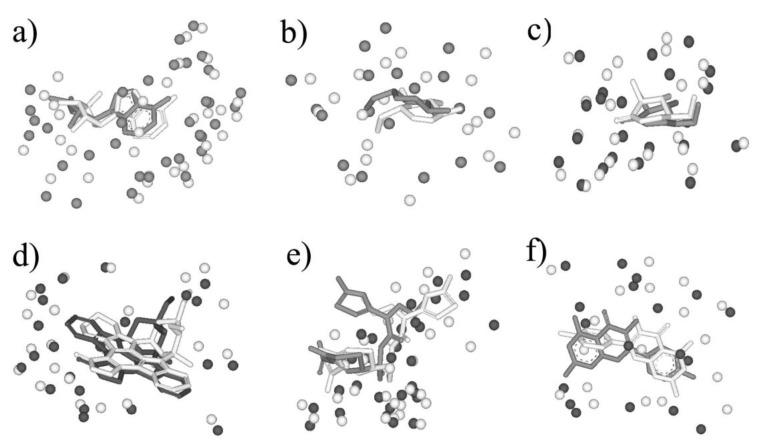
(**a**) The matched binding sites for 5′-deoxyadenosine between Glutamate mutase (in white, PDB entry: 1I9C) and Methylmalonyl-coamutase (in black, PDB entry: 4REQ) by BSMM (Sequence Identity: 7%); (**b**) the matched binding sites for 1,5-anhydrosorbitol between D-xylose isomerase (in white, PDB entry: 1XIE) and Maltodextrin phosphorylase (in black, PDB entry: 2ASV) by BSMM (Sequence Identity: 3%); (**c**) the matched binding sites for isofagomine lactam between β-Glucosidase (in white, PDB entry: 1UZ1) and β-Mannosidase (in black, PDB entry: 2VJX) by BSMM (Sequence Identity: 11%); (**d**) the matched binding sites for Staurosporine between CDK2 (in white, PDB entry: 1AQ1) and Phosphatidylinositol 3 kinase (in black, PDB entry: 1E8Z) by BSMM (Sequence Identity: 9%); (**e**) the matched binding sites for Cetotaxime group between D-alanyl-d-alanine carboxypeptidase (in white, PDB entry: 1CEF) and Toho-1 beta-lactamase (in black, PDB entry: 1IYO) by BSMM (Sequence Identity: 7%); (**f**) the matched binding sites for Kaempherol between Quercetin 2,3-dioxygenase (in white, PDB entry: 1H1M) and Udp-glucose flavonoid 3-o glycosyltransferase (in black, PDB entry: 2C1Z) by BSMM (Sequence Identity: 6%).

Six pairs were shown as the examples in Figure 5, in which the pair sequence identity is no more than 12% and the Mscore is more than 0.5. 5′-deoxyadenosine (5AD) binds both Glutamate mutase (PDB entry: 1I9C) and Methylmalonyl-coamutase (PDB entry: 4REQ), and the sequence identity of the two proteins is 7%. The matching by BSMM for the binding sites of the pair gives the high score 0.729 with the RMSD value 1.16, which means the two binding sites are highly similar, and ligand 5AD binds the two sites in a similar mode (Figure 5a). Although the sequence identity of D-xylose isomerase and Maltodextrin phosphorylase is 3%, it shows that the binding sites of 1,5-anhydrosorbitol (ASO) for D-xylose isomerase and Maltodextrin phosphorylase also match well, with a matching score of 0.528 and an RMSD value of 1.47 (Figure 5b). For β-Glucosidase and β-Mannosidase, the sequence identity is 7%, the enzyme active sites are highly similar with an Mscore of 0.742, and the inhibitor isofagomine lactam binds the two sites in similar mode with an RMSD of 0.95 (Figure 5c). Furthermore, for kinase CDK2 (PDB-ID: 1AQ1) and PI3K (PDB-ID: 1E8Z), the global folding is largely different, and the sequence identity is 9%. The ATP active sites of them match well by BSMM with an Mscore of 0.606 and an RMSD of 2.55 (Figure 5d). For the cetotaxime group (CEF), its binding sites for D-alanyl-d-alanine carboxypeptidase and Maltodextrin phosphorylase match well with an Mscore of 0.714 (Figure 5e); however, the RMSD value is 3.69, due to the different conformation of the side chain. While, for the binding sites of Quercetin 2,3-dioxygenase and Udp-glucose flavonoid 3-o-glycosyltransferase pairs, which match well by BSMM with an Mscore of 0.553, the RMSD value of the ligand is 6.47 and the ligands are matched head to tail (Figure 5f). 

### 3.2. Similar Site

**Figure 6:**
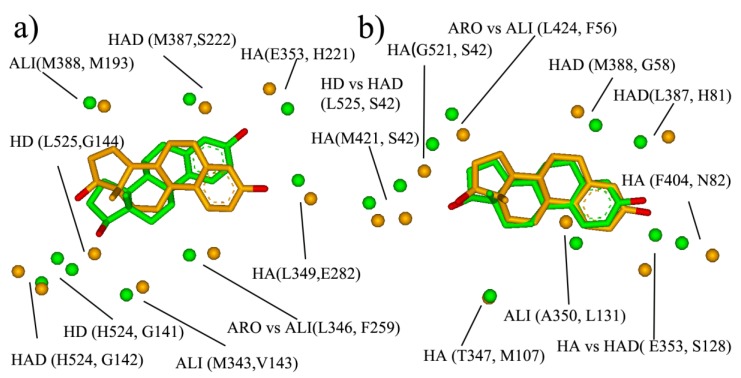
The similar sites and subsites: (**a**) the similar site of ER (in green, PDB entry: 1GWR) and 17- β-HSD (in orange, PDB entry: 1IOL); (**b**) the similar site of ER (in green, PDB entry: 1GWR) and sex hormone binding globulin (in orange, PDB entry: 1LHU).

Most current multi-target ligand design is in ligand-based strategy through the combination of selective ligands. In such cases, the designed multi-target ligand usually has a large molecular size and the further optimization of the initial multi-target ligand remains a huge challenge. Target-based strategies—such as using similar binding-sites—might provide another approach for the design of the multi-target ligands. The similar site will be outputted after BSMM matching, which consists of the physicochemical property points of the hydrophobic (ALI, ARO) hydrogen bond (HA, HD, HAD), and may provide rather useful information for the multiple ligands design, such as the pre-filter pharmacophore model before large-scale docking. Two examples are shown here to describe the similar binding site, and the results are shown in Figure 6.

**Figure 7:**
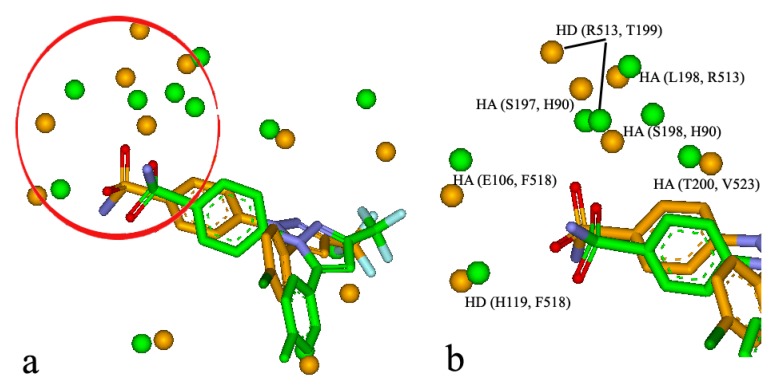
(**a**) The similar site of CAII (in green, PDB entry: 1OQ5) and that of COX-2 (in orange, PDB entry: 6COX); (**b**) the similar subsite of CAII and COX-2 taken from.

The first example is the pair of ER and 17- β-HSD1 (Figure 6a). ER and 17- β-HSD1 play important roles in breast cancer. Two-thirds of breast cancers are hormone-dependent, in that their growth is governed largely by interactions between estrogen and estrogen receptors. 17HSD1 predominantly catalyzes the reduction in estrone to estradiol. ER-positive patients that received tamoxifen and had 17HSD1 amplification showed decreased breast cancer survival rates. The estrogen receptor selective estrogen receptor modulators (SERM), tamoxifen is widely used to treat early and advanced ER-positive breast cancer, and the development of the 17HSD1 inhibitor has been a focus for many research groups with promising results in pre-clinical studies. Therefore, the ligands that could inhibit both ER and 17HSD1 activity might be a promising therapeutic agent for breast cancer. Here, the Estradiol bind-sites of 17HSD1 (1IOL) and ER (1GWR) were matched by BSMM with the matched score of 0.375 and the RMSD of 2.83 Å, and the result was shown in Figure 7a. The similar site is constituted by ARO vs. ALI (ER: L346 vs. 17- β-HSD: F259), ALI vs. ALI (ER: M343 vs. 17- β-HSD: V143), HD vs. HD (ER: L525 vs. 17- β-HSD: G144), HD vs. HD (ER: H524 vs. 17- β-HSD: G141), HAD vs. HAD (ER: H524 vs. 17- β-HSD: S142), ALI vs. ALI (ER: L388 vs. 17- β-HSD: M193), HA vs. HA (ER: G353 vs. 17- β-HSD: H221), and HA vs. HA (ER: L349 vs. 17- β-HSD: E282), which involve five hydrogen bond interactions and three hydrophobic interactions, and would provide sufficient interaction points for the new ligand design.

The other example is the pair of ER and SHBG (Sex hormone-binding globulin). Sex hormone-binding globulin, the specific carrier for sex steroids, regulates the bioavailable hormone fraction and estrogen signaling system in breast cancer cells. It inhibits estradiol-induced cell proliferation. Moreover, the protein also inhibits estradiol anti-apoptotic effect, by blocking ERK ½ activation elicited by the estradiol membrane-initiated pathway. Therefore, the ER antagonist which does not bind with SHBG should be the more promising therapeutic agent for ER-positive breast cancer. The BSMM-matching of two binding sites gave a matched score of 0.490 and an RMSD of 1.16 Å. The similar site of ER and sex hormone-binding globulin (Figure 6b) is constituted by ALI vs. ALI (ER: A350 vs. SHBG: L131), HAD vs. HA (ER: E353 vs. SHBG: S131), ALI vs. ARO (ER: I424 vs. SHBG: F56), and HD vs. HAD (ER: L525 vs. SHBG: S42). According to biological knowledge, it is the ligand which could inhibit both ER and 17HSD1 activity, but not interact with SHBG will exhibit better potency. Comparing the similar site of ER and SHBG with that of ER and 17HSD1, it is interesting that the two similar sites have a large difference, although the binding modes of the ligand estradiol are similar. Specifically, the physicochemical points which are involved in the similar site of ER and SHBG but not in that of ER and 17HSD1 are important for the multiple-ligand design to combat ER-positive breast cancer.

### 3.3. Similar Subsite

It is well-known that some binding sites are composed by multiple sub-sites, which play an important role in biological function and ligand binding. For the BSMM matching, the similar subsites were obtained by identifying the largest family from a cluster analysis of the matched points of the matched binding sites. For example, it was reported that celecoxib, a COX-2 inhibitor, has unexpected nanomolar inhibition potency against carbonic anhydrase II (CAII) (9). The crystal structure of the CAII in complex with celecoxib has been resolved (PDB entry: 1OQ5). The crystal structure of COX-2 in complex with the analogue of celecoxib was also solved (PDB entry: 6COX). BSMM gave a match score of 0.469 for the two complexes and the matched results are shown in Figure 7a,b. The two ligands bind to the two binding sites in a similar mode. Specifically, the matched similar sites consist of ALI vs. ALI (CAII: V135 vs. COX-2: L359), HD vs. HD (CAII: P202 vs. COX-2: R120), ARO vs. ALI (CAII: F131 vs. COX-2: V349), ALI vs. ALI (CAII: V121 vs. COX-2: A516), HA vs. HA (CAII: E106 vs. COX-2: F518), HAD vs. HD (CAII: H119 vs. COX-2: F518), HD vs. HD (CAII: S197 vs. COX-2: H90), HD vs. HD (CAII: T199 vs. COX-2: R513), HD vs. HAD (CAII: L198 vs. COX-2: R513), and HA vs. HA (CAII: H94 vs. COX-2: L352). A similar subsite was detected in the binding sites of CAII and COX-2 and is shown in Figure 7b. This similar subsite consisted of HA vs. HA (CAII: E106 vs. COX-2: F518), HAD vs. HD (CAII: H119 vs. COX-2: F518), HD vs. HD (CAII: S197 vs. COX-2: H90), and HA vs. HA (CAII: H94 vs. COX-2: L352). This subsite has been identified as the binding pocket for the phenyl sulfonamide functional group, and the subsimilar site would help the further optimization of the phenyl sulfonamide functional group.

### 3.4. Limitation and Future Development of BSMM

Based on geometric hashing algorithms and represention of a binding-site with PC points, current version of BSMM software is able to detect proteins with similar binding sites or subsites, which could be of great value for the design of multi-target ligands. However, there are still a few challenges remains, future improvements are highly required. Firstly, the flexibility of binding site plays critical role in ligang/receptor recognition. Therefore the flexibility is important issue in protein binding site matching. In current version, we do not consider the unique flexibility of each binding site residue which is supposed to be significantly different. We plan to consider the more reasonable flexibility parameter using the alignment of crystal structures of same protein to check the displacement of the binding site residue (both the back-bone and the side-chain). According to the displacement value, we may define flexibility potential (FP) as a important parameter in future binding site matching. Besides the protein flexibility, the flexibility of ligand is also important in ligand/receptor recognization. In some cases, the ligand binds to the different targets in different binding modes, and the current version BSMM could not consider the flexibility of the ligand. Another drawback for BSMM is that current similarity score function does not consider the weight of the PC points during the matching. For the contribution of each PC point to the binding of ligand is different., a weight parameter of each PC point should be needed during the binding site matching. What is more, current outputted similar site or similar subsite is inconvenient to be applied in drug design, and more friendly interface of transformable file format should be considered. Otherwise, the binding affinity is rather important issue in structure based drug design and development. The binding affinity prediction would be the option of further development of BSMM. A combination of BSMM with the reversed docking strategy would greatly help the multiple ligand design.

## 4. Conclusions

Based on geometric hashing algorithms and the representation of a binding-site with PC points, we have developed the BSMM software to detect proteins with similar binding sites or subsites. In particular, BSMM is independent of protein global folds and sequence and is therefore applicable to the matching of any binding sites. The similar sites between protein pairs with low homology and/or different folds are generally not obvious to visual inspection. The detection of such similar binding sites by BSMM could be of great value for the design of multi-target ligands. Further development of BSMM will focus on the refinement of similarity score functions and the incorporation of the binding site flexibility into the matching process. 

## Figures and Tables

**Table 1:** Matching rule of the physicochemical points.

PC Point	The Matching PC Point
HA	HA, HAD
HD	HD, HAD
HAD	HA, HD, HAD
ALI	ALI, ARO
ARO	ALI, ARO

## Supplementary Materials

The supplementary materials are available online.
