# Supplementary material for: Binding-Site Match Maker (BSMM): A Computational Method for the Design of Multi-Target Ligands
Source: Molecules. 2020 Apr 16;25(8):1821. doi: 10.3390/molecules25081821 (PMC7221819; doi:10.3390/molecules25081821)
Supplement: Supplementary file 1 [file molecules-25-01821-s001.pdf]

Supplementary Materials: Binding-site Match Maker (BSMM): A Computational Method for the Design of Multi-Target Ligands

Jinming Zhou,[a,b*] and Jian Hui Wu[c-e,*]

[a]Key Laboratory of the Ministry of Education for Advanced Catalysis Materials, Department of Chemistry, Zhejiang Normal University, 688 Yingbin Road, Jinhua 321004, P. R. China [b]Drug discovery & innovation center, College of Chemistry and Life Sciences, Zhejiang Normal University, 688 Yingbin Road, Jinhua 321004, P. R. China [c]Segal Cancer Center; [d]Lady Davis Institute for Medical Research, Sir Mortimer B. Davis-Jewish General Hospital, McGill University, 3755 Cote-Ste-Catherine, Rd., Montreal, QC H3T 1E2, Canada; and [e]Department of Oncology, McGill University

Supplementary Materials

Appendix 1 Description of the binding site 2

Appendix 2 The algorithm of the geometric hashing 4

Table S1. The matched binding sites from proteins with similar global folds 7

Table S2. The matched binding sites from proteins with different global folds (Set B). 11

Matched binding-site....................11

Appendix 1   Description of the binding site

a) The atom name in the pdb file:

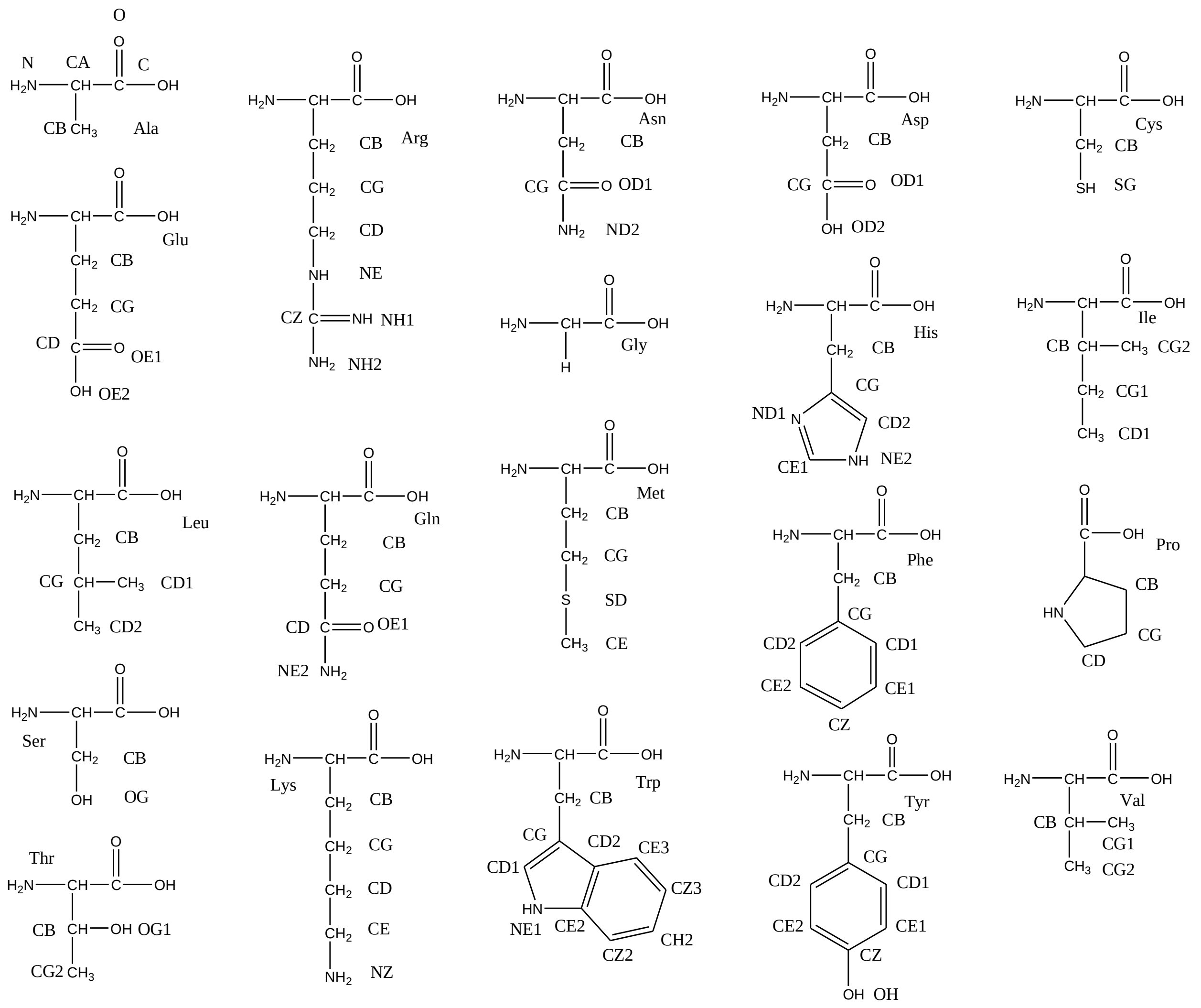

b) The table of how to calculate the coordinates of Pctype (physicochemical) points, and the **mass center** of the involved atoms was adopted as the coordinates of the ALI and ARO type points.

Residue type	Pctype and Atoms for calculating PCtype
Ala	ALI (CB)
Val	ALI (CG1, CG2)
Ile	ALI (CG1, CG2, CD1)
Leu	ALI (CD1, CD2)

Met	ALI (SD, CE)
Phe	ARO (CG, CD1, CD2, CE1, CE2, CZ)
Tyr	ARO(CG, CD1, CD2, CE1, CE2, CZ);<br>HAD (OH)
Trp	ARO (CD2, CE2, CE3, CZ2, CZ3, CH2);<br>HAD (NE1)
Lys	HD(NZ)
Arg	HD (NH1, NH2)
His	HD (NE2); HAD(ND1)
Thr	HAD (OG1)
Ser	HAD (OG)
Gln	HAD (OE1, OE2)
Asn	HAD (ND2, OD1)
Glu	HA (OE1, OE2)
Asp	HA (OD1, OD2)
Pro	/
Gly	/
Cys	SH (SG)

Appendix 2 The algorithm of the geometric hashing

Built a new coordinate (Q) based on the three points (P) p1(x1, y1, z1), p2(x2, y2, z2), p3(x3, y3, z3): p1 is as the origin point (0, 0, 0); vector p2-p1 serves as the x-axis (1, 0, 0); and the y-axis (0,1,0) is the vector p2-p1 turn a 90 degree in the (p1, p2, p3) plane; the z-axis (0, 0, 1) is defined as the right hand rule.

Math:

Point: p1(x1, y1, z1), p2(x2, y2, z2), p3(x3, y3, z3);

Vector: p2-p1 $\begin{matrix} x2-x1 \\ y2-y1 \\ z2-z1 \end{matrix}$ p3-p1 $\begin{matrix} x3-x1 \\ y3-y1 \\ z3-z1 \end{matrix}$

x-axis of the new coordinate:

$$X = (p2-p1)/|p2-p1| = A \times \begin{bmatrix} 1 \\ 0 \\ 0 \end{bmatrix}$$

z-axis:

$$Z = (p2-p1)\times(p3-p1)/|(p2-p1)\times(p3-p1)| = A \times \begin{bmatrix} 0 \\ 0 \\ 1 \end{bmatrix}$$

y-axis:

$$Y = Z \times X = A \times \begin{bmatrix} 0 \\ 1 \\ 0 \end{bmatrix}$$

X, Y, Z is the 3×1 Matrix, while A is the 3×1 Matrix. It is easy to know that *A* may be (*X Y Z*).

Therefore, the transform Matrix is mostly like A^{-1}

For any points in the new coordinate q = A^{-1} (p-p1)

Matrix calculation:

$P_1 = a_1 i + b_1 j + c_1 k \qquad Q_1 = a_2 i + b_2 j + c_2 k$

Then:

$$| P_1 | = \sqrt{a_1^{\,2} + b_1^{\,2} + c_1^{\,2}}$$

$$P_1 \times Q_1 = \begin{vmatrix} b_1 & c_1 \\ b_2 & c_2 \end{vmatrix} i - \begin{vmatrix} a_1 & c_1 \\ a_2 & c_2 \end{vmatrix} j + \begin{vmatrix} a_1 & b_1 \\ a_2 & b_2 \end{vmatrix} k$$

$$A = \begin{bmatrix} a_{11} & a_{12} & a_{13} \\ a_{21} & a_{22} & a_{23} \\ a_{31} & a_{32} & a_{33} \end{bmatrix}$$

Then:

$$A^{-1} = \frac{1}{|A|}\begin{bmatrix} \begin{vmatrix} a_{22} & a_{23} \\ a_{32} & a_{33} \end{vmatrix} & \begin{vmatrix} a_{13} & a_{12} \\ a_{33} & a_{32} \end{vmatrix} & \begin{vmatrix} a_{12} & a_{13} \\ a_{22} & a_{23} \end{vmatrix} \\ \begin{vmatrix} a_{23} & a_{21} \\ a_{33} & a_{31} \end{vmatrix} & \begin{vmatrix} a_{11} & a_{13} \\ a_{31} & a_{33} \end{vmatrix} & \begin{vmatrix} a_{13} & a_{11} \\ a_{23} & a_{21} \end{vmatrix} \\ \begin{vmatrix} a_{21} & a_{22} \\ a_{31} & a_{32} \end{vmatrix} & \begin{vmatrix} a_{12} & a_{11} \\ a_{32} & a_{31} \end{vmatrix} & \begin{vmatrix} a_{11} & a_{12} \\ a_{21} & a_{22} \end{vmatrix} \end{bmatrix}.$$

Example:

The p1 (1, 1, 0), p2 (2, 2, 0), p3(0, 2, 0)

The calculation of *X*, *Y*, *Z*

$$X = \begin{bmatrix} \frac{\sqrt{2}}{2} \\ \frac{\sqrt{2}}{2} \\ 0 \end{bmatrix} \quad Y = \begin{bmatrix} -\frac{\sqrt{2}}{2} \\ \frac{\sqrt{2}}{2} \\ 0 \end{bmatrix} \quad Z = \begin{bmatrix} 0 \\ 0 \\ 1 \end{bmatrix} \text{ and } \quad A = \begin{bmatrix} \frac{\sqrt{2}}{2} & -\frac{\sqrt{2}}{2} & 0 \\ \frac{\sqrt{2}}{2} & \frac{\sqrt{2}}{2} & 0 \\ 0 & 0 & 1 \end{bmatrix}$$

Then $A^{-1} = \begin{bmatrix} \frac{\sqrt{2}}{2} & \frac{\sqrt{2}}{2} & 0 \\ -\frac{\sqrt{2}}{2} & \frac{\sqrt{2}}{2} & 0 \\ 0 & 0 & 1 \end{bmatrix}$ a simple check: the p (0, 0, 0) should be $(-\sqrt{2},0,0)$

in the new coordinates, q = A^{-1}((0,0,0)-(1,1,1)), the result is $(-\sqrt{2},0,0)$.

Table S1. **The matched binding sites from proteins with similar global folds**

Ligand	Reference binding-site		Matched binding-site		Mscore	RMSD (Å)	Sequence identity (Q-score)
	Protein	PDB-ID	Protein	PDB-ID			
CGP 53820	HIV-1 protease	1HIH	HIV-2 PROTEASE	1HII	0.943	0.23	48%（0.91）
N~2~,n~2~-dimethyl-n~1~-(6-oxo-5,6-dihydrophenanthridin-2-yl)glycinamide	Exotoxin A	1XK9	Cholix toxin	2Q6M	0.886	2.11	34%（0.65）
Olomoucine	Cyclin-dependent kinase 2	1W0X	Extracellular regulated kinase 2	4ERK	0.787	1.43	37%（0.38）
2-({6-[(3-chlorophenyl)amino]-9-isopropyl-9h-purin-2-yl}amino)-3-methylbutan-1-ol	Proto-oncogene tyrosine-protein kinase Src	1YOM	Casein kinase i isoform gamma-3	2IZU	0.683	1.77	19%（0.37）
Methyl 5,7-dihydroxy-2-methyl-4,6,11-trioxo-3,4,6,11-tetrahydrotetracene-1-carboxylate	Nogalonic acid methyl ester cyclase	1SJW	Aklanonic Acid methyl Ester Cyclase, aknh	2F98	0.702	7.53	66%（0.92）
(Z)-1h,1'h-[2,3']biindolylidene-3,2'-dione- 3-oxime	Glycogen synthase kinase-3 beta	1Q41	Cdc2-like CDK2/CDC28 like protein kinase	2QKR	0.756	1.17	31% (0.49)
			Cyclin-dependent kinase 5	1UNH	0.804	0.89	33%（0.54）
[4-(4-hydroxy-3-isopropylphenoxy)-3,5-dimethylphenyl]acetic acid	Hormone receptor alpha 1, THRA1	1NAV	Thyroid hormone receptor beta-1	1NAX	0.946	0.41	84% (0.85)
Glucoimidazole	Beta-galactos	2CEQ	Beta-glucosidase	2CES	0.969	0.34	28%

	idase						(0.58)
3-aminobenzoic acid	Ornithine aminotransferase	1GBN	Glutamate semialdehyde aminotransferase	3GSB	0.633	2.32	21% (0.55)
N-[4-(3-bromo-phenylamino)-quinazolin-6-yl]-acrylamide	Proto-oncogene tyrosine-protein kinase Src	2HWP	Epidermal growth factor receptor	2J5F	0.766	2.48	32% (0.56)
Carbenoxolone	3-alpha, 20 beta-hydroxy steroid dehydrogenase	1HDC	Corticosteroid 11-beta-dehydrogenase isozyme 1	2BEL	0.438	9.15	20% (0.59)
Aldosterone	Mineralocorticoid receptor	2AA2	Ancestral corticoid receptor	2Q1H	0.945	0.79	74% (0.90)
N-[2-(4,8-dioxo-1,3-dioxa-6-aza-2$l^{2}-cupracyclooct-6-yl)ethyl]-4-sulfamoyl-benzamide	Carbonic Anhydrase II	2FOV	Carbonic anhydrase I	2FOY	0.804	0.86	80% (0.88)
4-bromophenol	toluene, o-xylene monooxygenase oxygenase subunit	1T0S	Methane monooxygenase component A alpha chain	1XU3	0.704	2.16	19%（0.37)
2,7-bis-(4-amidinobenzylidene)-cycloheptan-1-one	Trypsin	1V2N	Tissue plasminogen activator	1A5H	0.788	1.98	40%（0.67）
AZ 242	Peroxisome proliferator activated receptor gamma	1I7I	Peroxisome proliferator activated receptor alpha	1I7G	0.778	1.55	58%（0.79）
N-acetyl-l-norvaline	Ornithine carbamoyltransferase	1ZQ8	putative ornithine carbamoyltransferase	2G7M	0.865	1.59	39% (0.63)

Fluorescein	Ig gamma-2A chain C region	1FLR	4m5.3 anti-fluorescein single chain antibody fragment	1X9Q	0.979	0.40	0.24 (0.531)
TO-901317	RXR-beta	1UHL	LXR-beta	1UPV	0.962	2.59	71% (0.78)
			PXR	2O9I	0.431	2.60	24% (0.61)
CRA_8696	Thrombin	1O2G	Beta-trypsin	1O3F	0.951	0.70	35% (0.74)
			Urokinase-type plasminogen activator	1O5A	0.941	0.53	28% (0.68)
Estradiol	ERα	1GWR	ERβ	2J7X	0.941	0.40	57% (0.83)
Diethylstilbestro l	ERRγ	1S9P	ERα	3ERD	0.875	0.56	32% (0.69)
Staurosporine	Cyclin-depen dent protein kinase 2 (CDK2)	1AQ1	c-Src	1BYG	0.921	0.59	20% （0.46）
			3-Phosphoinositide dependent protein kinase 1	1OKY	0.881	0.63	23% （0.46）
			Glycogen synthase kinase-3β	1Q3D	0.824	0.91	34% （0.44）
			LCK kinase	1QPD	0.795	0.67	20% 0.40
			CAMP-dependent protein kinase	1STC	0.756	1.05	21% （0.38）
			Death-associated protein kinase 1	1WVY	0.821	0.67	24% （0.44）
			Protein kinase C, theta type	1XJD	0.911	0.51	21% （0.45）
			Proto-oncogene serine/threonine-prot ein kinase Pim-1	1YHS	0.825	1.53	23% （0.44）
			Serine/threonine-prot ein kinase TAO2	2GCD	0.911	1.66	24% （0.41）
			EGFR	2ITQ	0.811	1.79	19% （0.33）
			Interleukin-1	2NRY	0.867	1.08	27%

			receptor-associated kinase 4				（0.37）
			MAP kinase-activated protein kinase 2	2PZY	0.818	0.83	21%（0.36）
			Ribosomal protein S6 kinase alpha-1	2Z7R	0.886	0.75	25（0.49）
			Tyrosine-protein kinase CSK	3D7T	0.811	0.58	21%（0.40）
Imatinib	c-Kit	1T46	Tyrosine-protein kinase SYK	1XBB	0.649	11.59	32%（0.58）
			Proto-oncogene tyrosine-protein kinase ABL1	2HYY	0.918	0.57	35%（0.71）
			Proto-oncogene tyrosine-protein kinase Src	2OIQ	0.917	1.18	33%（0.64）
			Proto-oncogene tyrosine-protein kinase LCK	2PL0	0.938	0.98	35%（0.51）

Table S2. **The matched binding sites from proteins with different global folds (Set B).**

**Ligand**	**Reference binding-site**		**Matched binding-site**		**M-score**	**RMSD (Å)**	**Q-Score (%)**
	**Protein**	**PDB-ID**	**Protein name**	**PDB-ID**			
Estradiol	ERα	1GWR	Estrogenic 17-β-Hydroxysteroid dehydrogenase 1	1IOL	0.375	2.83	4% （0.032）
			Sex hormone-binding Globulin	1LHU	0.490	1.16	3% （0.010）
			Sulfotransferase 1A1	2D06	0.409	3.10	8% （0.031）
DHT	Sex hormone-binding Globulin	1D2S	Estrogenic 17-β-Hydroxysteroid dehydrogenase	1DHT	0.628	2.87	3% （0.024）
Diethylstilbestrol	ERRγ	1S9P	Transthyretin	1TT6	0.643	2.92	7% （0.018）
Salicylic acid	Xanthine oxidase	1FIQ	Salicylic acid carboxyl methyltransferase	1M6E	0.619	2.08	7% （0.022）
			salicylate synthetase, Irp9	2FN1	0.615	2.21	7% （0.026）
			Serum albumin	2I2Z	0.483	2.93	6% （0.012）
			Transcriptional regulator	3BPX	0.483	3.91	12% （0.024）
1-anilino-8-naphthalene sulfonate	Udp-n-acetylglucosamine 1-carboxyvinyltransferase	1EYN	Pheromone binding protein	1OW4	0.422	3.72	4% （0.023）
			Adipocyte lipid-binding protein	2ANS	0.452	3.22	9% （0.019）
5'-deoxyadenosine	Glutamate mutase	1I9C	5'-fluoro-5'-deoxyadenosine synthase	2CC2	0.542	2.66	11% （0.093）
			Molybdenum cofactor biosynthesis protein a	2FB3	0.472	3.39	4% （0.078）
			Methylmalonyl-coa	4REQ	0.729	1.06	7%

			mutase				（0.17）
L-benzylsuccinic acid	Carboxypeptidase	1CBX	Thermolysin	1HYT	0.619	2.99	8%（0.041）
			Serine carboxypeptidase II	1WHT	0.512	2.38	7%（0.070）
Chloramphenicol	Type III chloramphenicol acetyltransferase	1CLA	Hth-type transcriptional regulator TTGR	2UXP	0.400	3.46	3%（0.038）
S-(p-nitrobenzyl)glutathione	Glutathione s-transferase YFYF	1GLQ	Ure2 protein	1K0C	0.630	3.99	5%（0.042）
Alpha-amylase	Alpha amylase, pancreatic	1U2Y	Alpha-mannosidase 2	3D51	0.529	2.16	4%（0.022）
Cholesterol	Beta-elicitin cryptogein	1LRI	Beta-elicitin cryptogein	1ZHY	0.364	3.32	6%（0.015）
Isopropyl-1-beta-d-thiogalactoside	Beta-galactosidase	1JYX	Galactoside o-acetyltransferase	1KRU	0.412	3.39	8%（0.0086）
Staurosporine	Cyclin-dependent protein kinase 2	1AQ1	Phosphatidylinositol 3–kinase	1E8Z	0.606	2.55	9%（0.025）
Naringenin	Chalcone synthase	1CGK	Hth-type transcriptional regulator TTGR	2UXU	0.556	2.56	1%（0.019）
5-amino-5-deoxy-cellobiono-1,5-lactam	Oxidoreductase	1NAA	glucooligosaccharide oxidase	2AXR	0.333	2.33	2%（0.019）
4-(2-aminoethyl) benzenesulfonyl fluoride	Glyceraldehyde-3-phosphate dehydrogenase	2B4R	V-type ATP synthase beta chain	3B2Q	0.381	9.51	8%（0.028）
Aminomethylcyclohexane	Udp-n-acetylglucosamine enolpyruvyl	1DLG	Trypsin	1TNG	0.833	1.56	6%（0.017）

	transferase mura						
5'-o-[(s)-{[(5s)-5-amino-6-oxohexyl]amino}(hydroxy)phosphoryl]adenosine	Hypothetical 37.6 kda protein in gp24-hoc intergenic region	2HVQ	Putative DNA ligase-like protein Rv0938/MT0965	1VS0	0.762	1.58	8%（0.12）
4-nitro-2-phenoxymethanesulfonanilide	Phospholipase A2 VRV-PL-viiia	1ZWP	Lactotransferrin'	3E9X	0.600	5.70	11%（0.029）
Naphthalen-1-yl-acetic acid	Auxin-binding protein	1LRH	SKP1-like protein 1A	2P1O	0.517	2.11	6%（0.029）
Merck-kgaa-emd56133	Endothiapepsin	1E80	Acetolactate synthase, mitochondrial	1T9B	0.615	8.73	11% NA
Phenyl boronic acid	Cocaine esterase	1JU3	Trypsin	2A32	0.462	4.71	2%（0.019）
D-glucosamine	Ym1 secretory protein	1E9L	Exo-beta-d-glucosaminidase	2VZS	0.406	5.35	8%（0.041）
N~2~,n~2~-dimethyl-n~1~-(6-oxo-5,6-dihydrophenanthridin-2-yl)glycinamide	Exotoxin A	1XK9	Poly [ADP-ribose] polymerase 3	3CE0	0.818	0.96	5%（0.084）
Metyrapone	Cytochrome p450-cam	1PHG	Cytochrome p450 3a4	1W0G	0.536	5.18	11%（0.28）
3,5,7-trihydroxy-2-(3,4,5-trihydroxyphenyl)-4h-chromen-4-one	Phosphatidylinositol 3-kinase catalytic subunit	1E90	Dihydroflavonol 4-reductase	2IOD	0.388	7.08	10%（0.0097）
			Proto-oncogene serine/threonine-protein kinase Pim-1	2O63	0.562	3.84	9%（0.027）
4-methylcatechol	Catechol 1,2-dioxygenase	1DMH	Nicotinate-nucleotide--dimethylbenzimidazole phosphoribosyltransferase	1L4G	0.591	3.30	9% 0.018）
2-amino-4-trifluoromethylsulfan	Methionine aminopeptid	1C22	Methionyl-trna synthetase	1PFW	0.400	2.04	10%（0.0096）

yl-butyric acid	ase						
L-dopamine	Monoamine-sulfating phenol sulfotransferase	2A3R	Ribosyldihydronicotinamide dehydrogenase	2QMZ	0.357	3.52	4%（0.045）
			S-norcoclaurine synthase	2VQ5	0.500	4.67	7%（0.017）
			Phenylalanine 4-monooxygenase	5PAH	0.529	5.14	6% 0.024）
(R)-n-(2-methylbenzyl)-3-[(2s,3s)-2-hydroxy-3-(3-hydroxy-2-methylbenzoyl)amino-4-phenylbutanoyl]-5,5-dimethyl-1,3-thiazolidine-4-carboxamide	HIV protease	1KZK	Plasmodium malariae	2ANL	0.721	6.61	11%（0.12）
Kaempherol	Quercetin 2,3-dioxygenase	1H1M	Udp-glucose flavonoid 3-o glycosyltransferase	2C1Z	0.553	6.47	6%（0.0096）
Iso-ursodeoxycholic acid	3-alpha-hydroxysteroid dehydrogenase	1IHI	Bile acid receptor	1OT7	0.400	8.56	6%（0.029）
4-(diazenylcarbonyl)pyridine	Arylamine n-acetyltransferase	1W6F	Actinorhodin polyketide ketoreductase	1XR3	0.481	4.61	7%（0.023）
			Cytochrome c peroxidase	2V2E	0.387	6.08	2%（0.027）
			Ascorbate peroxidase	2VCN	0.519	2.74	3%（0.027）
1,4-dideoxy-1,4-imino-1-(s)-(9-deazaguanin-9-yl)-d-ribitol	Purine nucleoside phosphorylase	1B8N	Guanine phosphoribosyltransferase	1DQP	0.6	3.86	5%（0.053）
D-[(amino)carbonyl]phenylalanine	Carboxypeptidase	1HDU	N-carbamyl-D-amino acid amidohydrolase	1UF8	0.526	5.10	8%（0.031）
(3s,4r,5r)-3,4-dihydroxy-5-(hydro	Beta-glucosidase	1UZ1	Man5a, mannosidase	1UZ4	0.742	0.95	7%（0.20）

xymethyl)piperidin- 2-one			Endoglucanase	2V38	0.581	1.46	14%（0.21）
			Beta-mannosidase	2VJX	0.774	0.85	11% 0.070）
s-benzyl-glutathione	Lactoylglutathione lyase	1FRO	Glutathione s-transferase a1-1	1GUH	0.366	6.91	4%（0.020）
(5r,6r,7s,8s)-3-(anilinomethyl)-5,6,7,8-tetrahydro-5-(hydroxymethyl)-imidazo[1,2-a]pyridine-6,7,8-triol	Beta-D-glucan exohydrolase isoenzyme exoi	1X39	Beta-glucosidase a	2J7C	0.438	4.19	8%（0.063）
3-(n-hydroxycarboxamido)-2-isobutylpropanoyl-trp-methylamide	Lethal factor	1PWU	Alpha-mannosidase 2	3D4Z	0.531	2.96	5%（0.0043）
			Catrocollastatin	2DW0	0.574	2.56	4%（0.014）
			Coagulation factor X-activating enzyme heavy chain	2E3X	0.569	3.41	6%（0.013）
			Vascular apoptosis-inducing protein 1	2ERP	0.509	2.49	1%（0.013）
3-aminobenzoic acid	Ornithine aminotransferase	1GBN	Poly [ADP-ribose] polymerase	2PQF	0.500	2.43	2%（0.021）
N1-carboxypiperazine	Cathepsin	1MEM	Signal recognition particle protein	1OKK	0.500	3.05	3%（0.043）
(2r,3r)-2-(3,4-dihydroxyphenyl)-3,5,7-trihydroxy-2,3-dihydro-4h-chromen-4-one	Leucoanthocyanidin dioxygenase	1GP5	Dihydroflavonol 4-reductase	2C29	0.486	7.49	5%（0.015）
Bishydroxy[2h-1-benzopyran-2-one,1,2-benzopyrone]	Oxygen-insensitive NAD(P)H nitroreductase	1OOQ	NAD(P)H dehydrogenase [quinone] 1	2F1O	0.357	4.70	5%（0.027）
2-deoxy-2-fluoro-b-d-cellobioside	Xylanase	1E0V	Endoglucanase	5A3H	0.606	3.08	5%（0.023）
2-[[3-(trifluorom	Transthyreti	1BM7	Aldo-keto reductase	1S2C	0.412	5.98	11%

ethyl)phenyl]amino] benzoic acid	n		family 1 member C3				（0.020）
Castanospermine	Exo-(b)-(1,3)-glucanase	1EQC	Beta-glucosidase a	2CBU	0.786	0.75	4%（0.058）
			Alpha-glucosidase	2JKP	0.607	2.67	10%（0.057）
			Sucrose isomerase	2PWG	0.607	3.42	7% 0.097）
N-[3-benzyl-5-(4-hydroxyphenyl) pyrazin-2-yl]-2-(4-hydroxyphenyl)acetamide	Obelin	2F8P	Renilla-luciferin 2-monooxygenase	2PSJ	0.447	5.21	2%（0.024）
Chymostatin	The peptide amidase PAM	1M21	Alkaline serine protease	1WVM	0.469	7.07	9%（0.019）
Flavopiridol	Glycogen phosphorylase	1C8K	Cell division protein kinase 9	3BLR	0.489	5.81	8%（0.0098）
Cefotaxime group	D-alanyl-d-alanine carboxypeptidase	1CEF	Penicillin-binding protein 1b	2UWY	0.673	5.14	6%（0.12）
Coumarin	Cytochrome P450, family 2, subfamily A, polypeptide 6	1Z10	Xenobiotic reductase A	2H90	0.474	2.01	5%（0.0088）
			Proteinase K	2PWB	0.304	1.44	4%（0.013）
			Lactotransferrin	3CRB	0.600	2.83	6%（0.0075）
1,5-anhydrosorbitol	D-xylose isomerase	1XIE	Maltodextrin phosphorylase	2ASV	0.528	1.47	3%（0.020）
			Coagulation factor VII	2EC9	0.400	5.81	/（0.019）
